# A Systematic Framework for Identifying Prognostic Genes in the Tumor Microenvironment of Colon Cancer

**DOI:** 10.3389/fonc.2022.899156

**Published:** 2022-05-19

**Authors:** Jinyang Liu, Yu Lan, Geng Tian, Jialiang Yang

**Affiliations:** ^1^Department of Sciences, Geneis Beijing Co., Ltd., Beijing, China; ^2^Department of Data Mining,Qingdao Geneis Institute of Big Data Mining and Precision Medicine, Qingdao, China; ^3^PhD Workstation, Chifeng Municipal Hospital, Chifeng, China

**Keywords:** colon cancer, tumor microenvironment, DEGs, prognostic genes, risk score, prognostic model

## Abstract

As one of the most common cancers of the digestive system, colon cancer is a predominant cause of cancer-related deaths worldwide. To investigate prognostic genes in the tumor microenvironment of colon cancer, we collected 461 colon adenocarcinoma (COAD) and 172 rectal adenocarcinoma (READ) samples from The Cancer Genome Atlas (TCGA) database, and calculated the stromal and immune scores of each sample. We demonstrated that stromal and immune scores were significantly associated with colon cancer stages. By analyzing differentially expressed genes (DEGs) between two stromal and immune score groups, we identified 952 common DEGs. The significantly enriched Gene Ontology (GO) and Kyoto Encyclopedia of Genes and Genomes (KEGG) terms for these DEGs were associated with T-cell activation, immune receptor activity, and cytokine–cytokine receptor interaction. Through univariate Cox regression analysis, we identified 22 prognostic genes. Furthermore, nine key prognostic genes, namely, *HOXC8*, *SRPX*, *CCL2*2, *CD72*, *IGLON5*, *SERPING1*, *PCOLCE2*, *FABP4*, and *ARL4C*, were identified using the LASSO Cox regression analysis. The risk score of each sample was calculated using the gene expression of the nine genes. Patients with high-risk scores had a poorer prognosis than those with low-risk scores. The prognostic model established with the nine-gene signature was able to effectively predict the outcome of colon cancer patients. Our findings may help in the clinical decisions and improve the prognosis for colon cancer.

## Introduction

Colon cancer is a common malignant tumor, ranking second among cancers in causing cancer-related deaths in the United States. Statistics from 2016 and 2017 estimated that approximately 147,950 individuals would be diagnosed with colon cancer in 2020, with 53,200 of these individuals dying from the disease (1–3). In China, colon cancer has the fifth highest incidence and mortality among all cancers (4). The cure and survival rates for colon cancer have increased because of early cancer screening and improvements in treatment (5, 6).

The tumor microenvironment (TME) is composed of tumor cells and surrounding immune cells, stromal cells, and extracellular matrices (ECMs) (7–11). Tumor cells can interact closely with their niche, with mesenchymal stromal cells playing a role in tumor cells escaping surveillance of the immune system (12, 13). Stromal cells promote tumor growth by overexpressing growth signals in cancer cells (14). There is growing evidence that the TME results in tumor progression by participating in multiple biological processes, including immune cell activation and recruitment, angiogenesis, and ECM remodeling (8, 15). Therapeutic strategies targeting the TME have emerged as a promising approach for cancer treatment in recent years (16, 17). Many studies have indicated that TME can affect a patient’s clinical outcome and response to therapy (18, 19). Tumor-infiltrating immune cells have been proven to significantly influence tumor progression and the efficacy of anti-tumor therapy (20).

The function of multiple cell types in the TME of colon cancer has been well elucidated. In addition to acting as a physical scaffolding for tumor cells, ECM also contributes to colon cancer cells adhesion, immune evasion, and metastasis (21). Tumor-associated neutrophils enhance invasiveness by influencing angiogenesis and response to vascular endothelial growth factor (VEGF) inhibition in colon cancer (22). Higher numbers of CD4+ T cells can improve survival and patient benefits (23), whereas infiltrated inefficient T cells can drive tumor immune resistance (24). Malignant cells may avoid immune surveillance by suppressing dendritic cells, and colon cancer stem cells can evolve into malignant cells by accumulating genetic and epigenetic alterations and interacting with the TME as well (25). In summary, the TME of colon cancer promotes a pro-inflammatory milieu, and therefore, anti-inflammatory agents can be used to treat colon cancer (26).

In this study, we explored the relationship between stromal and immune scores of colon cancer and clinical variables. We then identified nine key prognostic genes in the TME of colon cancer. We established a novel prognostic model of the nine-gene signature that effectively predicted the outcome of colon cancer patients.

## Materials and Methods

### Colon Cancer Data Collection From the TCGA Database and GEO Database

Gene expression data and corresponding clinical information of COAD and READ patients used in our study were downloaded from The Cancer Genome Atlas (TCGA) database (https://portal.gdc.cancer.gov/). Detailed clinical characterization of the patients was summarized in **Table 1**. The Gene Expression Omnibus (GEO) database [GSE39582 (*n* = 585)] was used to validate the relationship between the expression of nine key prognostic genes and the survival of colon cancer patients.

**Table 1:** Clinical characterizations of patients.

Clinicopathologic variables	Category	Count (%) (*n* = 633)
**Sex**	Female	294 (46.4)
	Male	335 (52.9)
	Unknown	4 (0.6)
**Age**	≤65	253 (39.9)
	>65	376 (59.3)
	Unknown	4 (0.6)
**Stage**	Stage I	109 (17.2)
	Stage II	229 (36.1)
	Stage III	181 (28.5)
	Stage IV	90 (14.2)
	Unknown	24 (3.7)
**Stage T**	T1	20 (3.1)
	T2	109 (17.2)
	T3	428 (67.6)
	T4	70 (11.0)
	Unknown	6 (0.9)
**Stage N**	N0	357 (56.3)
	N1	151 (23.8)
	N2	118 (18.6)
	Unknown	7 (1.1)
**Stage M**	M0	467 (73.7)
	M1	89 (14.0)
	Unknown	77 (12.1)
**Survival status**	Alive	499 (78.8)
	Death	130 (20.5)
	Unknown	4(0.6)

### Calculation of the Stromal and Immune Scores and Identification of DEGs

We calculated the immune and stromal scores in each tumor sample using the “estimate” R package, and the gene expression matrix of colon cancer patients from the TCGA database was used as input (27). Patients were subsequently separated into high-stromal and low-stromal score groups or high-immune and low-immune score groups based on the median scores, respectively. DEGs were identified using the “limma” R package, (FDR) <0.05 and |log2(fold change)| >1 as the cutoff values (28, 29). The “heatmap” R package was employed to display the expression level of the top 40 DEGs. The “VennDiagram” R package was used to display the overlapping genes (30).

### Enrichment Analysis of Intersection DEGs

To explore the potential functions and pathways of these intersection DEGs, Gene Ontology (GO) and Kyoto Encyclopedia of Genes and Genomes (KEGG) enrichment analyses were performed by using the “enrichplot” package and the “clusterProfiler” package (31), with the threshold set as *p*-value < 0.05.

### Identification of Key Prognostic Genes Within Intersection DEGs

Univariate Cox regression analysis was used for identifying the relationship between gene expression and overall survival (OS), tumor samples of patients were divided into a high-expression group and a low-expression group according to the median gene expression level, *p*-value < 0.05 was considered as the threshold, and 22 genes were identified as candidate prognostic genes. A least absolute shrinkage and selector operation (LASSO) algorithm was used to identify key prognostic genes with the “glmnet” R package (32). Lambda.min was the cutoff point at which the minimum mean cross-validated error occurs. Genes or indexes whose coefficient was not 0 at lambda.min were selected as key prognostic genes. The risk score of each sample was calculated using the following formula:


$$\text{risk score}=\sum_{i}^{n}Expi*Coefi$$

Coef indicated the coefficient of genes and Exp indicated the expression level of genes. All patients were grouped into the high-risk group and low-risk group based on the median risk score. The “SurvivalROC” of R package was used to display the performance of all prognostic factors to predict the survival of colon cancer patients.

### Statistical Analysis

The correlation analysis was performed using Spearman’s correlation analysis. Survival curves were compared using the Kaplan–Meier method and the log-rank test. Cox regression analysis was used to calculate hazard ratios (HRs) and 95% confidence intervals (CIs). All tests were two-sided, and a *p* < 0.05 was considered to indicate significance.

## Results

### Stromal and Immune Scores Were Markedly Related to Colon Cancer Stages

**Figure 1:**
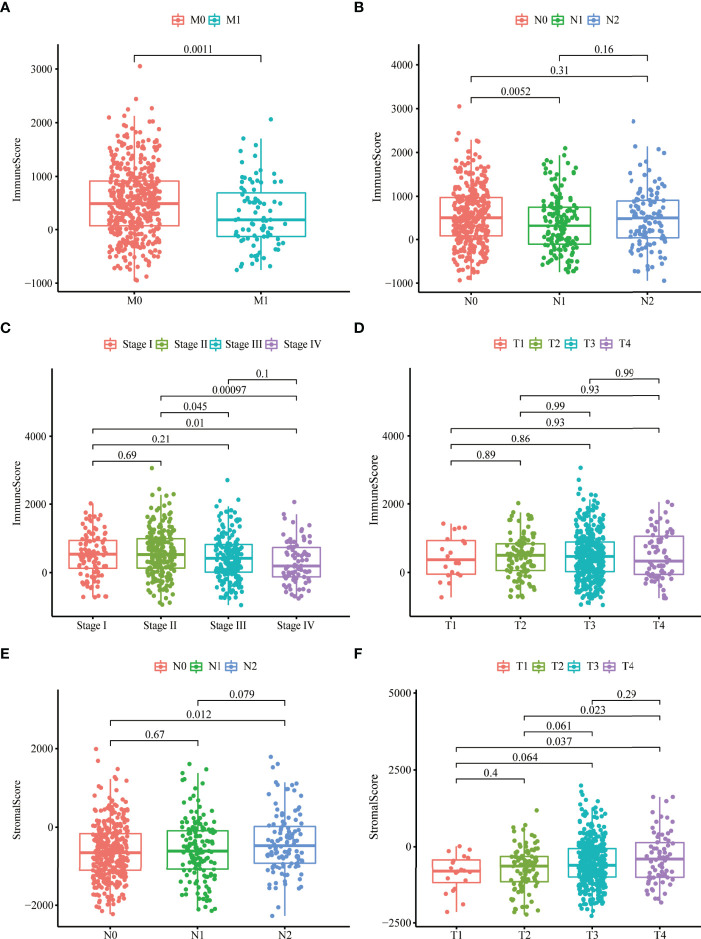
Stromal and immune scores were markedly related to colon cancer stages. **(A–D)** Distribution of immune scores in nonmetastatic (M0) patients and distant metastases (M1) patients **(A)**, N0-2 patients **(B)**, stage I–IV patients **(C)**, T1–4 patients **(D)**. **(E, F)** Distribution of stromal scores in N0-2 patients **(E)** and T1-4 patients **(F)**.

To investigate the relationship between stromal, immune scores, and clinical variables, we calculated the immune and stromal scores of each tumor sample. Patients with more malignant tumors exhibited lower immune scores than those with less malignant tumors (M1 vs. M0; N1 vs. N0; stage IV vs. stage I or stage II) (**Figures 1A–C**), whereas there were no differences in the distribution of immune scores among T1–4 patients (**Figure 1D**). We also observed no differences in the distribution of stromal scores among M0–1 patients or stage I–IV (**Supplementary Figures 1A, B**). The stromal scores for patients with more malignant tumors (N1 and T4) were higher compared to those with less malignant tumors (N0, T1, and T2) (**Figures 1E, F**). We did not observe significant associations between stromal scores or immune scores and age or sex (**Supplementary Figures 1C–F**).

### Identification of Intersection DEGs

**Figure 2:**
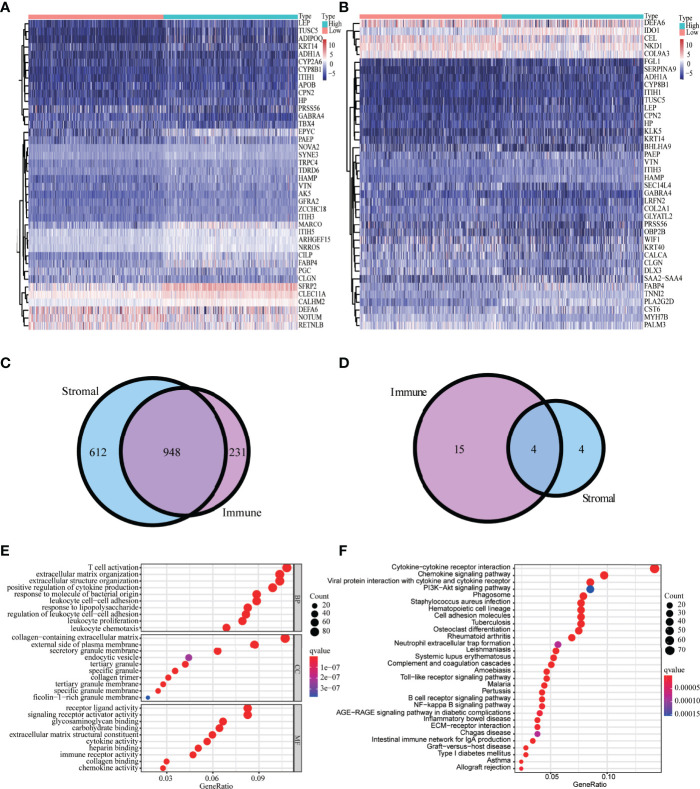
Analysis of DEGs-based stromal and immune scores. The heatmap of the top 40 DEGs based on stromal scores **(A)** and immune scores **(B)**. Venn diagrams displaying the number of upregulated DEGs **(C)** and downregulated DEGs **(D)** detected in both groups. Top 30 enriched (*p* < 0.05) GO terms **(E)** and KEGG terms **(F)**.

We identified 1,814 DEGs in high versus low immune and high versus low stromal score groups. The heatmap showed the gene expressions of the top 40 DEGs based on stromal scores and the top 40 DEGs based on immune scores, respectively (**Figures 2A, B**). We identified 948 common upregulated DEGs (**Figure 2C**) and four common downregulated DEGs (**Figure 2D**). GO enrichment analyses demonstrated that the main enriched terms for these intersection DEGs were T-cell activation, positive regulation of cytokine production, and immune receptor activity (**Figure 2E**). The significantly enriched KEGG terms were chemokine signaling pathway and cytokine–cytokine receptor interaction (**Figure 2F**).

### Identification of Key Prognostic Genes

**Figure 3:**
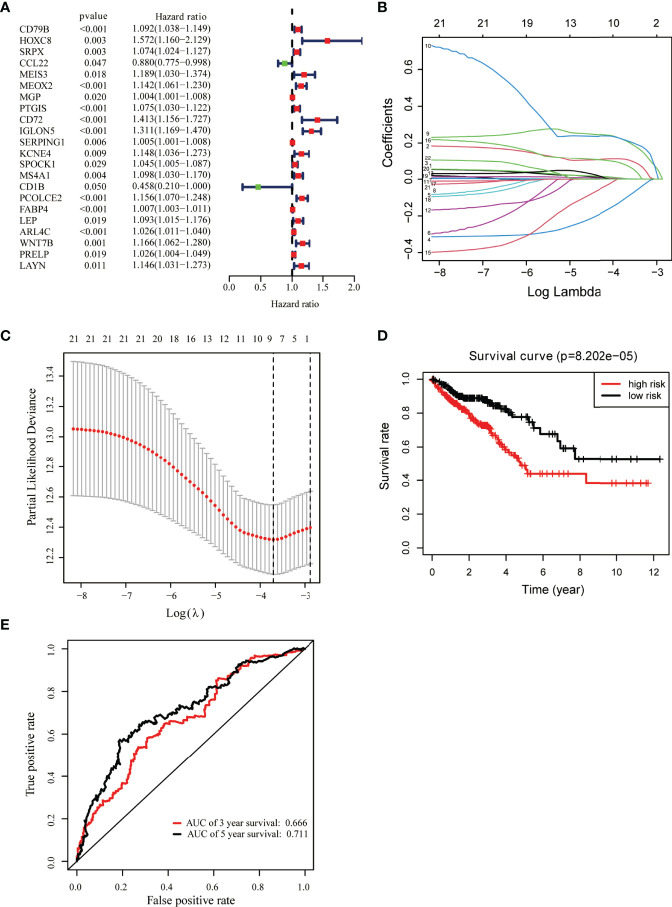
Identification of key prognostic genes within intersection DEGs. **(A)** Forest plot of risk genes: Red represented high-risk genes (hazard ratios, HR > 1); green represented low-risk genes (HR < 1). **(B)** Constructing the LASSO coefficient prediction model. **(C)** Selecting variables in LASSO regression with minimum criteria by 1,000 times cross-validation. **(D)** Overall survival between high- and low-risk score groups. **(E)** ROC curves for predicting 3- and 5-year overall survival probability with the nine-gene score.

Univariate Cox regression analysis was used for exploring the relationship between gene expression and OS (33). We identified 22 candidate prognostic genes, including 20 high-risk genes and two low-risk genes (**Figure 3A**). LASSO Cox regression analysis was used to identify key prognostic genes and build a model that can predict the prognosis of colon cancer patients (**Figures 3B, C**); we obtained nine key prognostic genes (**Table 2**). The OS between the low- and high-risk groups classified by our prognostic model was significantly different (*p* = 8.202e−05, **Figure 3D**). Next, we constructed the prognostic risk model with the nine-gene signature to predict 3- and 5-year OS; the area under the curve (AUC) of ROC curves of 3 and 5 years were 0.666 and 0.711, respectively (**Figure 3E**). To explore the correlation between the nine-gene risk score and TME score, we performed the Spearman’s correlation test, and the results showed that the nine-gene risk score was significantly correlated with the stromal or immune scores (**Supplementary Figures 2A, B**).

**Table 2:** Nine key prognostic genes.

Gene	Coef
CCL22	−0.103078
FABP4	0.000222
ARL4C	0.000224
SERPING1	0.000982
SRPX	0.002037
PCOLCE2	0.077076
HOXC8	0.108125
CD72	0.209473
IGLON5	0.211910

### Prognostic Genes Influenced the Proportion of Infiltrating Immune Cells

**Figure 4:**
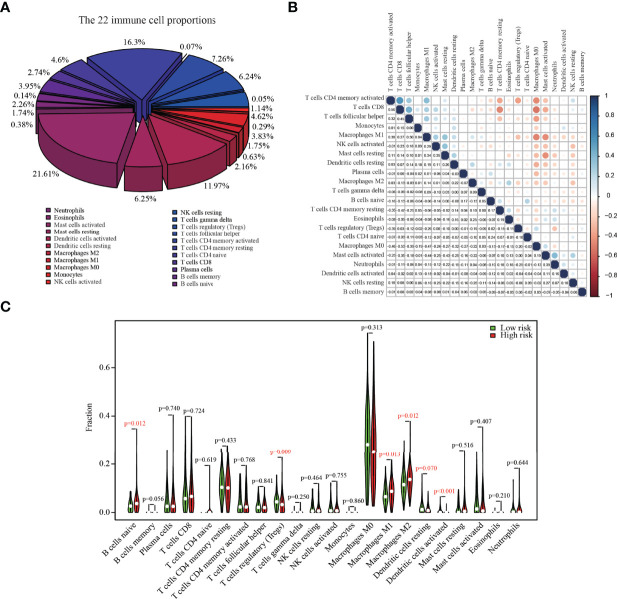
The composition of 22 immune cells in colon cancer tumors from the TCGA dataset. **(A)** The relative abundances of 22 immune cells in the tumor tissue of colon cancer patients. **(B)** The correlation matrix between different cell types; the size of the circle represented the degree of correlation. **(C)** Fractions of infiltrating immune cells in high versus low risk score groups.

The relative abundances of 22 immune cells in the tumor tissue of colon cancer patients are shown in **Figure 4A**, with M0 macrophages (21.61%), CD4+ resting memory T cells (16.29%), and M2 macrophages (11.97%) being the primary contributors to immune cell infiltration. CD8+ T cells exhibited a positive correlation with CD4+ memory T cells and a negative correlation with M0 macrophages (**Figure 4B**). The infiltration proportion of naïve B cells, M1 macrophages, and M2 macrophages was higher in high-risk score groups versus low-risk score groups, whereas the low-risk group had higher regulatory T cells (Tregs) (*p* = 0.009) (**Figure 4C**).

### Validation in the GEO Database

**Figure 5:**
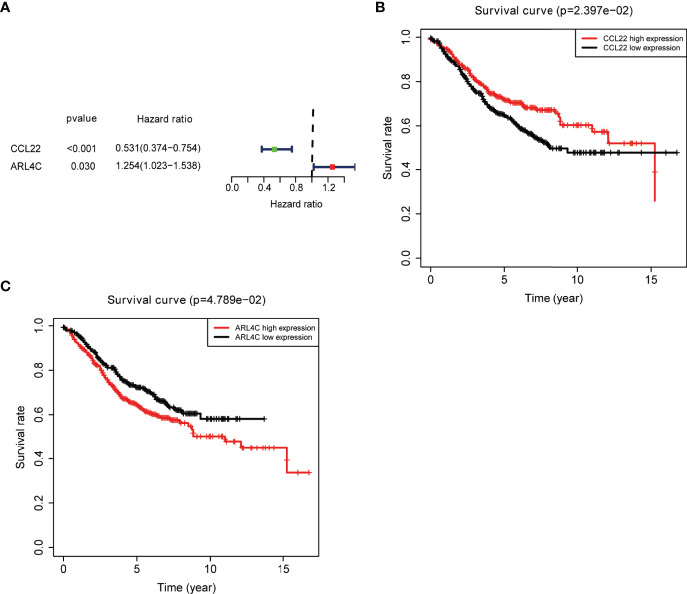
External validation of key prognostic genes using the GEO database. **(A)** Forest plot of risk genes: Red represents high-risk genes (hazard ratios, HR > 1); green represents low-risk genes (HR < 1). **(B, C)** Overall survival between high and low *CCL22* **(B)** and *ARL4C* **(C)** expression groups.

An external colon cancer dataset from the GEO database (GSE39582) was used to validate the correlation between the expression of the nine key prognostic genes and OS. Survival analysis was performed, and only two genes were matched in the dataset, including *CCL22* and *ARL4C* (**Figures 5A–C**).

## Discussion

TME was related to the development and progression of tumors and had the potential to influence responses to therapies. We obtained the immune and stromal scores that could reflect the degree of immune infiltration of corresponding cells in tumor tissue. We confirmed that stromal and immune scores were significantly related to colon cancer stages. Patients with more malignant tumors (M1, N1, and stage IV) have lower immune scores than those with less malignant tumors (M0, N0, stage I, and stage II); in contrast, the stromal scores for late-stage (N2 and T4) patients were higher compared to early-stage (N0, T1, and T2) patients. In the early stage of tumorigenesis, the TME of colon cancer was remodeled, the number of infiltrating stromal cells was raised, and the number of immune cells was decreased. Stromal cells helped tumor cells escape from being attacked by the immune system, and the lethality of some immune cells to tumors began to weaken. Disrupting the stability of TME thus induced tumor development.

We obtained DEGs between high versus low stromal and immune score groups and further identified 948 co-upregulated DEGs and four co-downregulated DEGs. The main enriched GO terms for the intersection DEGs were T-cell activation and ECM organization. Additionally, the significantly enriched KEGG terms were chemokine signaling pathway, and cytokine–cytokine receptor interaction.

Moreover, univariate Cox regression analysis was performed to determine the association between the expression of DEGs and survival, and we screened out 22 risk genes as candidate prognostic factors; using LASSO Cox regression analysis, we identified nine key prognostic genes. These genes have previously been reported to be associated with the development and progression of tumors. Fatty acid-binding protein 4 (*FABP4*) released from adipocytes could promote invasion in prostate cancer (34). ADP-ribosylation factor (Arf)-like protein 4c (*Arl4c*) expression was upregulated upon activation of Wnt-β-catenin and growth factor-Ras signaling and contributed to tubulogenesis and tumorigenesis (35). Serine proteinase inhibitor family G1 (*SERPING1*) downregulation was associated with poor prognosis in prostate cancer (PCa) (36). *SRPX2* was involved in tumor suppression and progression (37). Homeobox C8 (*HOXC8*) was a transcription factor that had been reported, and high expression of *HOXC8* was associated with poor prognosis of cervical cancer (38). Several types of immune cells, such as dendritic cells and macrophages, secreted *CCL22* upon activation (39–41). *CCL22* could recruit T regulatory cells and controlled the growth of tumor cells in melanoma. However, the relationship between *CCL22* and colon cancer was unknown. The level of chemokine *CCL22* was increased in COAD (42). Our study demonstrated that all these genes might be crucial biomarkers in the TME of colon cancer. We also found that *ARL4C* and *HOXC8* were upregulated, and *FABP4, PCOLCE2, SERPING1*, and *SRPX* were downregulated in tumor tissue compared to corresponding healthy tissue (**Supplementary Figure 3**). More work is needed to be done to investigate the association between the expression of these genes and colon cancer proliferation, metastasis, and invasion. We calculated the risk score of each sample using the gene expression of the nine genes, and we demonstrated that patients with high-risk scores have a poorer prognosis than those with a lower-risk score. Furthermore, we established an independent prognostic model that was able to effectively predict the outcome of colon cancer patients with the nine-gene signature.

Finally, analysis of immune cells’ infiltration revealed the M0 macrophages, CD4+ resting memory T cells, and M2 macrophages with the highest proportion. Many studies had shown that M2 macrophages may promote tumor progression (43), invasiveness (44), and angiogenesis (45). We also demonstrated that the infiltration proportion of M2 macrophages was higher in high-risk score groups versus low-risk score groups.

Because our study was a pure bioinformatics analysis based on the TCGA database, further biological experiments were needed to validate our results. Moreover, whether these nine key prognostic genes could improve the diagnostic accuracy and therapeutic response for colon cancer in actual clinical practice requires further verification.

## Conclusion

In conclusion, we identified nine potential prognostic markers for colon cancer through a systematic bioinformatics analysis. A novel prognostic model established with the nine-gene signature effectively predicted the outcome of colon cancer patients. More work is needed to validate our findings.

## Data Availability Statement

Publicly available datasets were analyzed in this study. These data can be found here: https://github.com/bensteven2/TME.

## Author Contributions

JY contributed to the conception and design of the study. JL performed the statistical and bioinformatic analysis. JL and YL wrote the manuscript with the help of JY. GT provided suggestions for figure preparation. All authors contributed to revising the manuscript, and read and approved the submitted version.

## Conflict of Interest

All authors were employed by Geneis Beijing Co., Ltd.

## Publisher’s Note

All claims expressed in this article are solely those of the authors and do not necessarily represent those of their affiliated organizations, or those of the publisher, the editors and the reviewers. Any product that may be evaluated in this article, or claim that may be made by its manufacturer, is not guaranteed or endorsed by the publisher.

## Supplementary Material

Supplementary Figure 1 Relationship between stromal and immune scores and colon cancer clinical variables. **(A, B)** Distribution of stromal scores in nonmetastatic (M0) patients and distant metastases (M1) patients **(A)**. Stage I-IV patients **(B)**. **(C–F)** Distribution of immune scores **(C)** and stromal scores **(D)** in different age groups, immune scores **(E)**, and stromal scores **(F)** in different sex groups.

Supplementary Figure 2 The correlation between the nine gene score and tumor microenvironment scores. **(A)** Stromal score; **(B)** Immune score.

Supplementary Figure 3 Comparison of the expressions of the nine key prognostic genes in tumor tissue and corresponding healthy tissue of colon cancer patients.
