# Supplementary figures and images for: A Systematic Framework for Identifying Prognostic Genes in the Tumor Microenvironment of Colon Cancer
Source: Front Oncol. 2022 May 19;12:899156. doi: 10.3389/fonc.2022.899156 (PMC9161737; doi:10.3389/fonc.2022.899156)

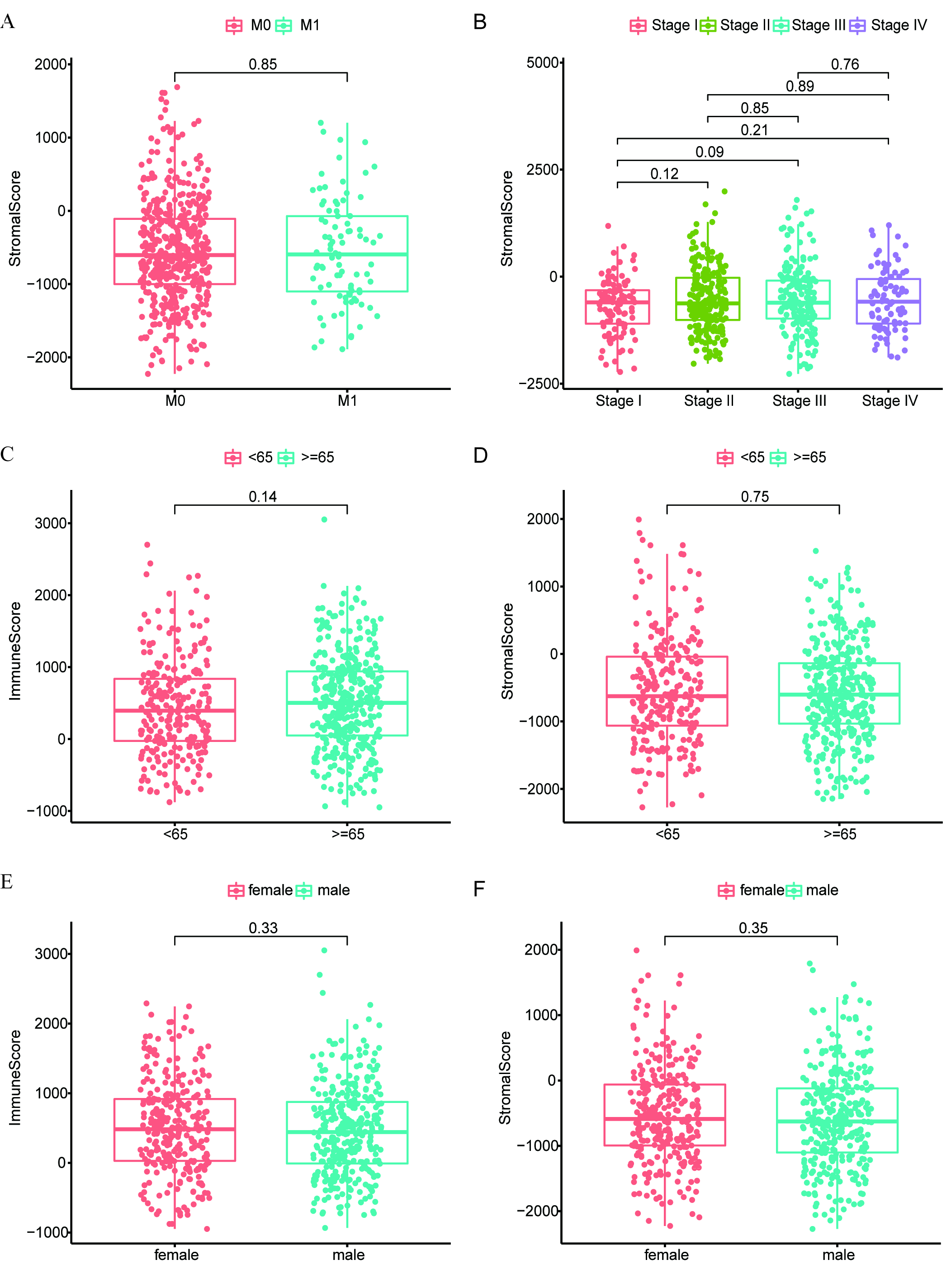

Supplement: Supplementary Figure 1 — Relationship between stromal and immune scores and colon cancer clinical variables. (A, B) Distribution of stromal scores in nonmetastatic (M0) patients and distant metastases (M1) patients (A). Stage I-IV patients (B). (C–F) Distribution of immune scores (C) and stromal scores (D) in different age groups, immune scores (E), and stromal scores (F) in different sex groups. [file Image_1.tif]

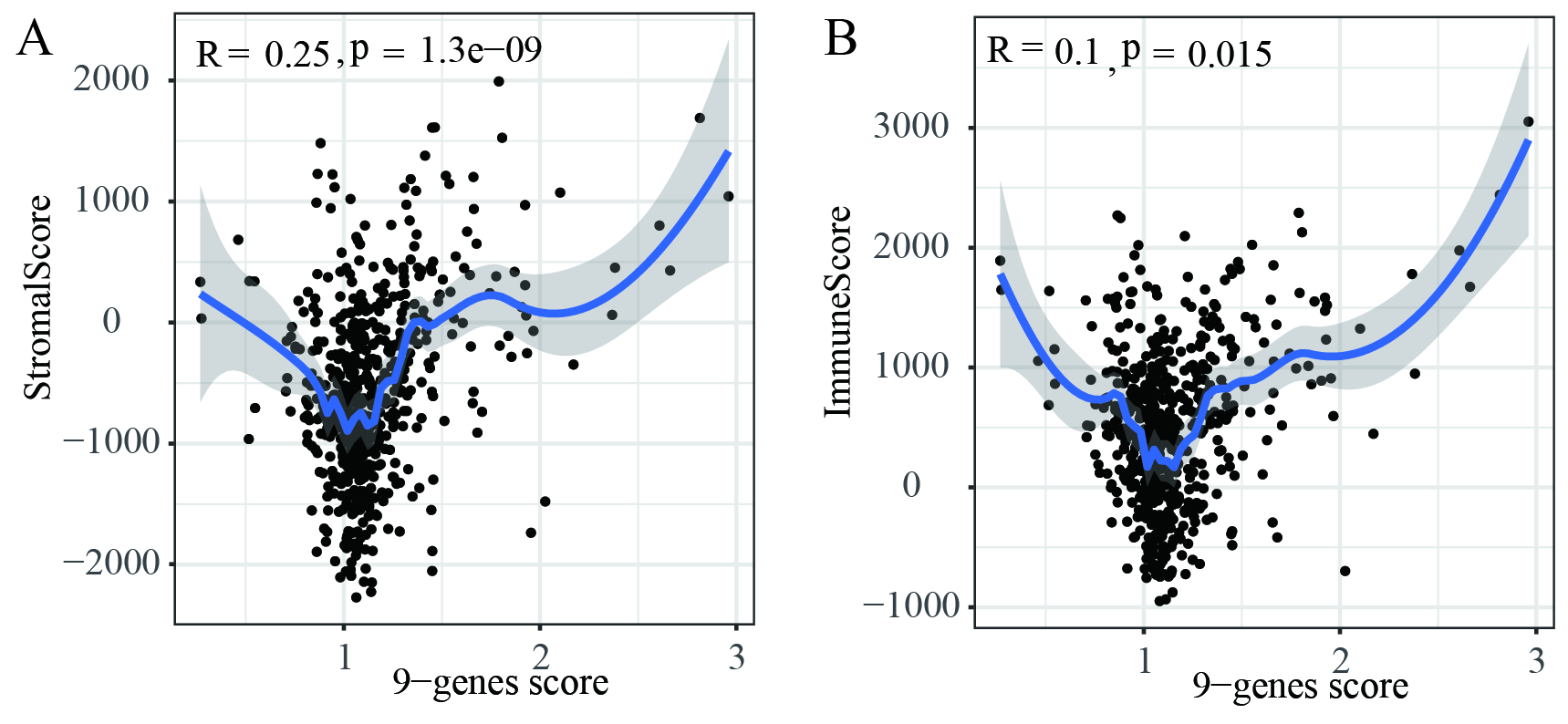

Supplement: Supplementary Figure 2 — The correlation between the nine gene score and tumor microenvironment scores. (A) Stromal score; (B) Immune score. [file Image_2.tif]

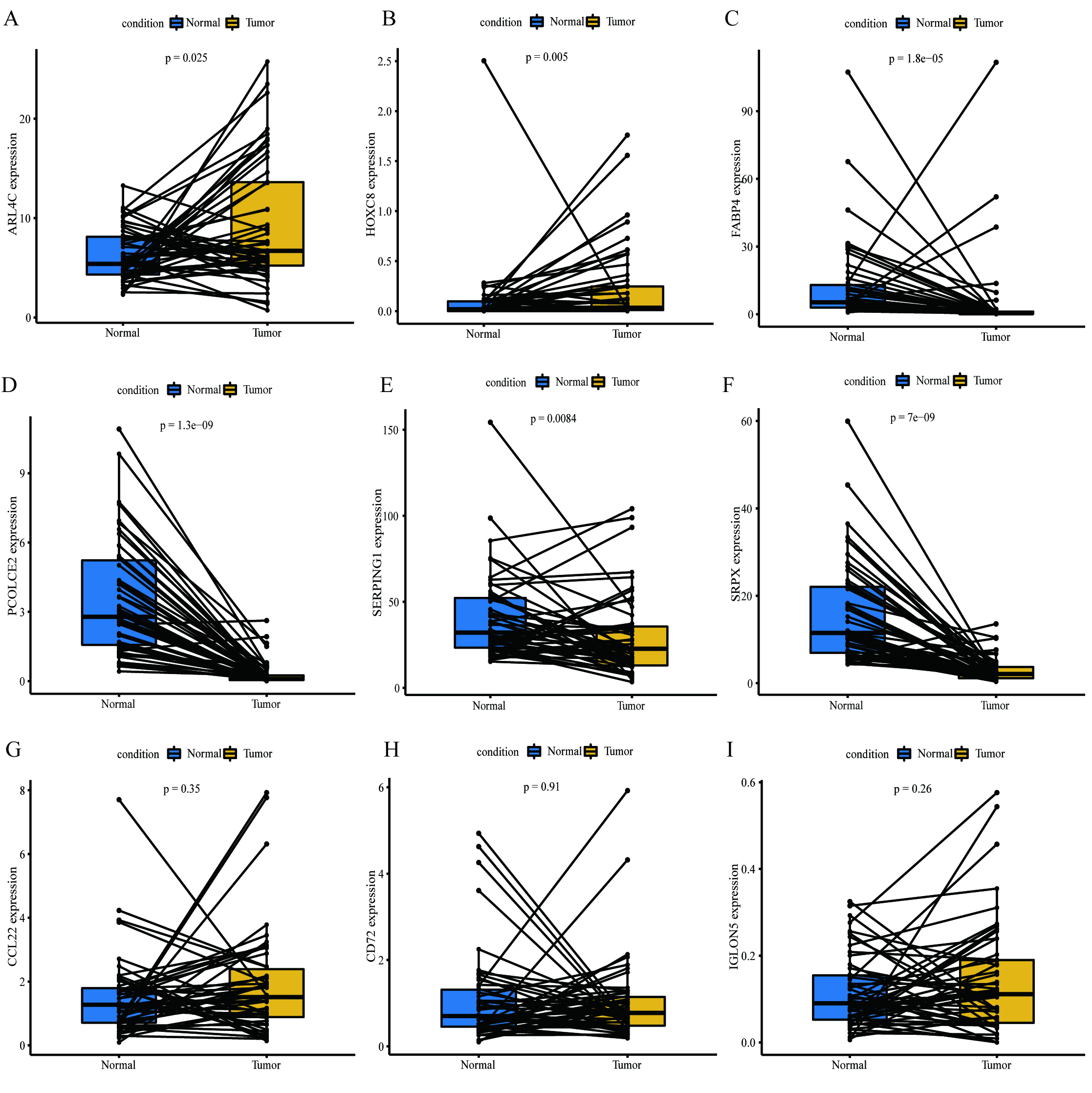

Supplement: Supplementary Figure 3 — Comparison of the expressions of the nine key prognostic genes in tumor tissue and corresponding healthy tissue of colon cancer patients. [file Image_3.tif]
